# Semi-prone thoracoscopic esophagectomy for esophageal carcinoma with aberrant right subclavian artery and non-recurrent inferior laryngeal nerve

**DOI:** 10.1186/s13019-022-01829-3

**Published:** 2022-04-23

**Authors:** Kazunori Koyama, Toru Watanabe, Hideaki Kato, Masahiko Kawaguchi

**Affiliations:** Department of General Surgery, Yokohama Sakae Kyosai Hospital, 132 Katsura-cho, Sakae-ku, Yokohama City, Kanagawa 247-8512 Japan

**Keywords:** Aberrant right subclavian artery, Esophageal carcinoma, Non-recurrent inferior laryngeal nerve, Semi-prone, Thoracoscopic esophagectomy

## Abstract

**Background:**

Aberrant right subclavian artery (ARSA) accompanied by non-recurrent inferior laryngeal nerve (NRILN) is a rare anomaly. In cases of thoracic esophageal carcinoma associated with ARSA and NRILN, surgeons must take extra care not to injury these vessels and nerves. We believe semi-prone thoracoscopic esophagectomy to be a surgical approach that can safely deal with such an anomaly.

**Case presentation:**

A 70-year-old man complained of feelings of chest constriction. Endoscopic examination revealed an esophageal tumor and computed tomography showed an ARSA. We performed semi-prone thoracoscopic esophagectomy for case with ARSA and NRILN. We identified these anomalies during esophagectomy, and we could complete surgery without injury these vessels and nerves. The patient had an uneventful recovery and discharged 22 days after surgery.

**Conclusions:**

Semi-prone thoracoscopic esophagectomy for esophageal carcinoma can be performed safely with a wide operative field, and is an excellent procedure for dissecting esophageal carcinoma in patients with ARSA and NRILN.

## Background

An aberrant right subclavian artery (ARSA) is a rare anomaly, often accompanied by non-recurrent inferior laryngeal nerve (NRILN), which causes a much higher risk of vessel and nerve injury and concomitant severe adverse events during esophagectomy, including bleeding, hoarseness, and pulmonary complications. We consider semi-prone thoracoscopic esophagectomy to be useful in such cases. The semi-prone position leads to fewer pulmonary complications and blood loss, decrease workload, and provide better ergonomic results in comparison with the left lateral decubitus position. Also, if required, conversion to a thoracotomy in the left decubitus position or supine position is easier from the semi-prone than from the prone position. Here, we report a semi-prone thoracoscopic esophagectomy performed to treat esophageal carcinoma with ARSA and NRILN.

## Case presentation

**Fig. 1:**
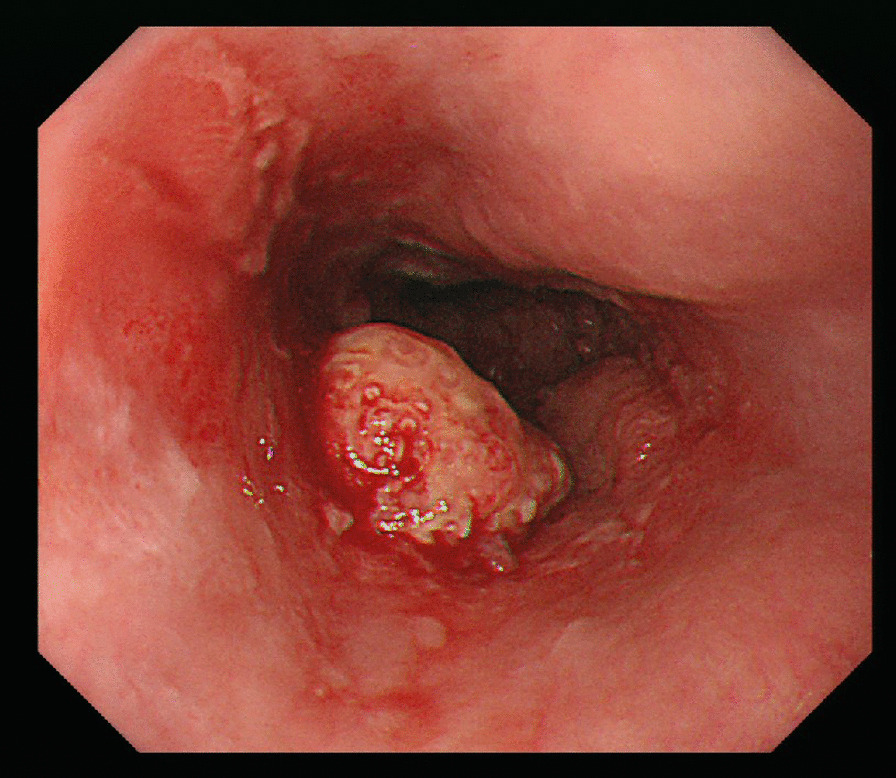
Photograph taken during endoscopic examination showing type 1 esophageal carcinoma extending from the mid to lower thorax

**Fig. 2:**
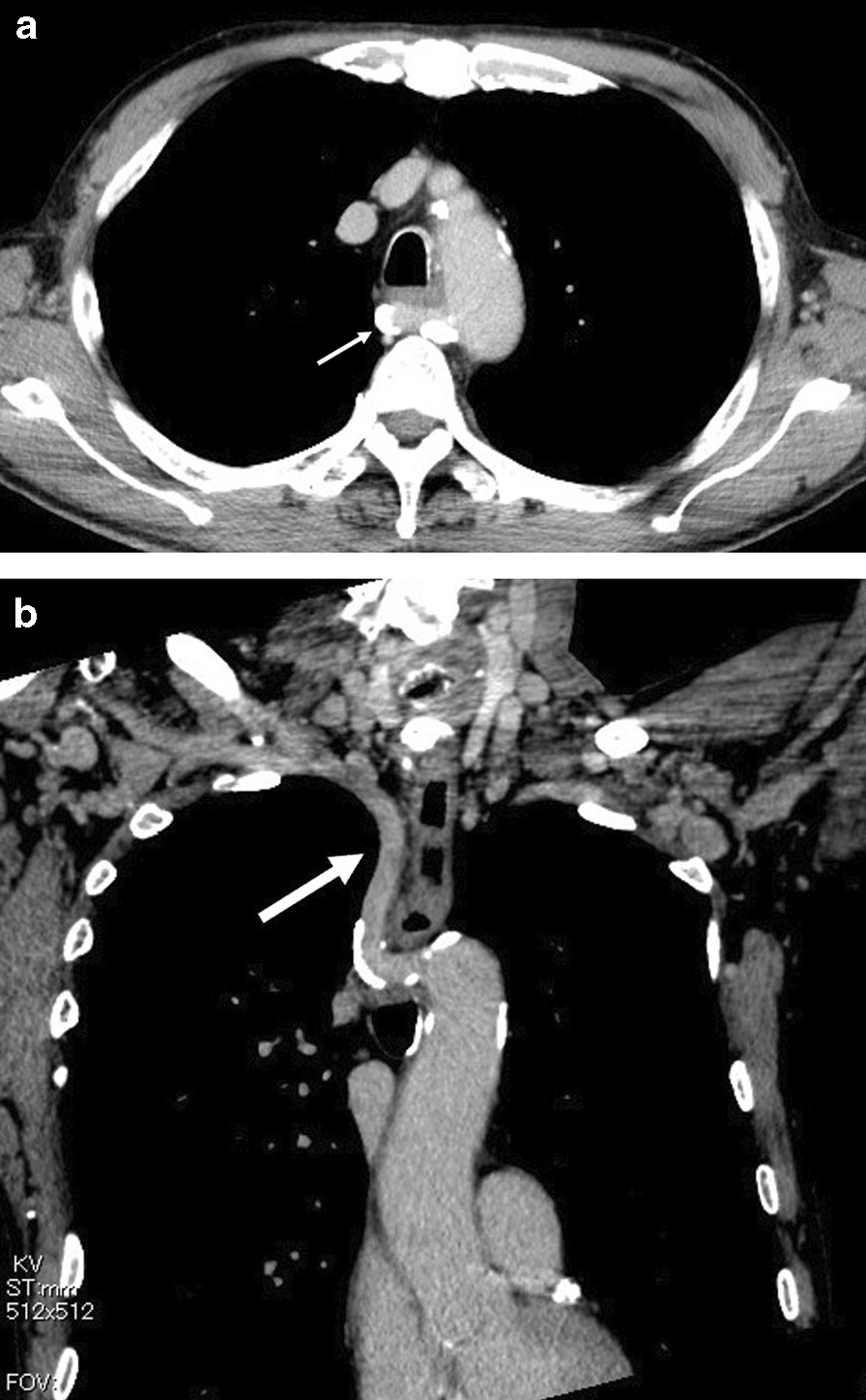
Computed tomographic image showing the aberrant right subclavian artery (arrow). **a** Transverse plane. **b** Coronal plane

**Fig. 3:**
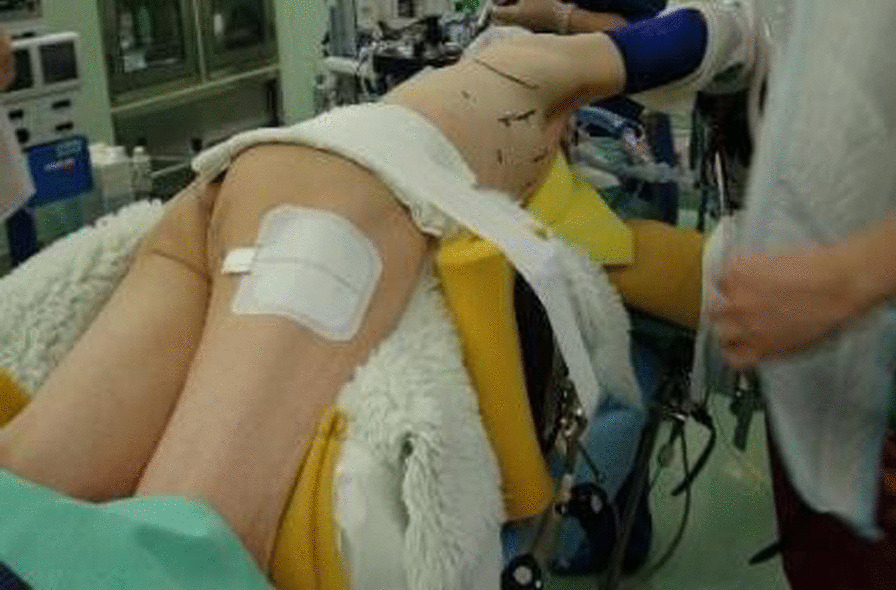
Photograph showing the patient in the semi-prone position

**Fig. 4:**
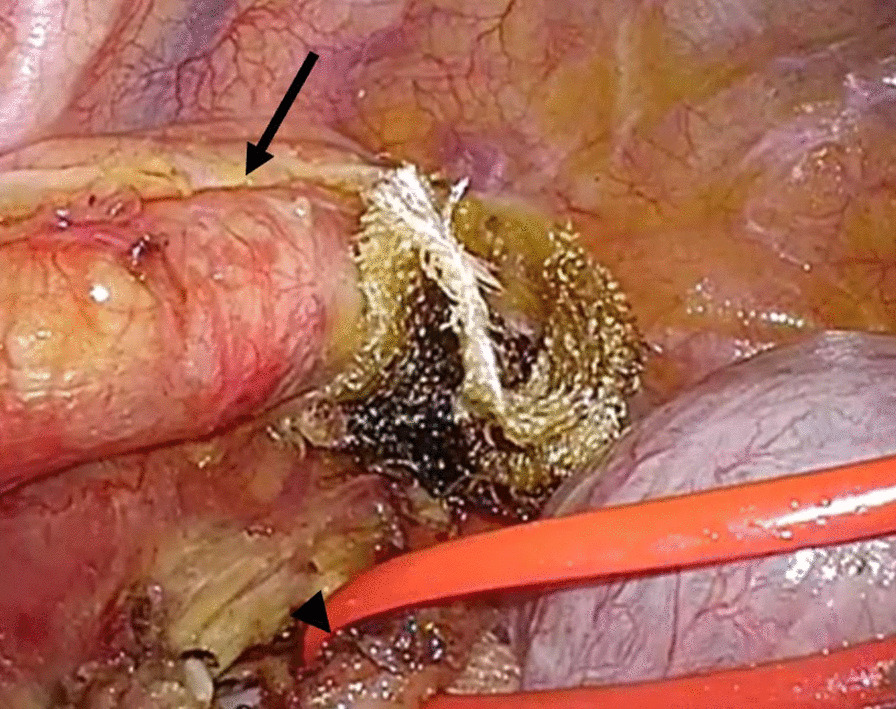
Intraoperative photograph showing the aberrant right subclavian artery (arrow) and the right vagus nerve (arrowhead)

A 70-year-old man complained of feelings of chest constriction. He was diagnosed as having esophageal cancer by means of endoscopy and was then hospitalized for surgery. Endoscopic examination showed type 1 esophageal carcinoma in the mid-thoracic esophagus (Fig. 1). Contrast-enhanced computed tomography revealed esophageal carcinoma in the mid-esophagus, an ARSA branching from the descending aorta (Fig. 2a, b), no enlargement of regional lymph nodes, and no distant metastasis. These findings led to the diagnosis of esophageal carcinoma, and with tumor-node-metastasis classification of cT1N0M0, c-Stage I. The patient had no history of smoking and alcohol consumption. He had a past medical history of diabetes mellitus. Semi-prone thoracoscopic esophagectomy was planned. We placed the patient in the left semi-lateral decubitus position with the right side of his body raised at an angle of 30° from the prone position and with the bed rotated ventrally. We placed ports in the third, fifth, and seventh intercostal spaces on the posterior axillary line (Fig. 3). After transecting the azygous arch and the right bronchial artery, the right subclavian artery (RSA) was found to branch directly from the descending aorta and travel along the left rear side of the esophagus. We taped the right vagus nerve prior to peeling the recurrent nerve from the esophagus (Fig. 4). Although the esophagus was positioned tightly between the trachea and the subclavian artery, we were able to resect the esophagus at a satisfactory location. Thoracoscopic esophagectomy and mediastinal lymph node dissection were then performed.

The patient was placed in the supine position, for commencement of abdominal surgery. The gastric tube conduit was pulled up through the retrosternal space, and end-to-end anastomosis was performed by hand-suturing in the neck field, preserving the NRILN. The operation time was 365 min and the intraoperative blood loss 150 ml. Pathological examination revealed moderately differentiated squamous cell carcinoma with no lymph node metastasis. The tumor had invaded the submucosa, with no apparent metastasis of dissected lymph nodes (pT1bN0M0; p-Stage I). The patient was taken to the intensive care unit while intubated and was subsequently extubated on postoperative day 1. The thoracic drainage tube was removed on postoperative day 7, and oral intake was begun on postoperative day 8. The patient’s postoperative course was uneventful, and he was discharged on postoperative day 22.

## Discussion and conclusions

ARSA is a congenital anomaly that develops from changes in anatomical development of the right fourth arch and the left sixth arch. The right fourth arch usually forms the RSA, and the left sixth arch forms an arterial duct. These vessels then descend into the thoracic cavity. When the right fourth arch disappears in early embryonic development, however, the RSA is formed from the right dorsal aorta and seventh intersegmental artery, and the RSA becomes the fourth branch of the aortic arch [1]. In such a case, because the resulting anatomy does not involve the right laryngeal nerve, the nerve branches from the vagal nerve trunk in the neck and becomes the NRILN [2, 3]. ARSA affects approximately 0.5–2% of the general population and is more frequent in women [4]; the frequency of NRILN associated with ARSA is 0.3–1.6% [3]. NRILN is not recognizable preoperatively. However, because enhanced computed tomography reveals the ARSA, the existence of NRILN is predictable [5].

Video-assisted thoracoscopic surgery (VATS) has been increasing in frequency worldwide because of minimally invasive surgical approaches and attempts to enlarge the visual field in esophageal cancer surgery. Three fields lymphadenectomy is important in radical surgery for esophageal cancer. VATS therefore provides a good surgical field of view for lymph-node dissection and enables a precise operation. In particular, VATS in the prone position enlarges the mediastinum by gravity, and leaking fluids and pooling blood do not interfere with the operative view, making esophagectomy safer [6–8]. Some reports have suggested that VATS is more technically difficult than open thoracotomy [3]. Moreover, if conversion to open surgery is necessary, it is difficult to change the body position when performing VATS on a patient in the prone position. Therefore, we employ the semi-prone position because it is easier to change the body position to left decubitus position or supine position and convert to open surgery than when the procedure is begun with the patient in prone position. Semi-prone position may overcome this problem with retaining the benefits of prone position.

Additionally, compared with the left lateral decubitus position, the semi-prone position provides a wide surgical field in the posterior mediastinum without lung retraction, and damage to the lung is comparably minimized. Because the semi-prone position eliminates the burden of requiring a skilled assistant, surgeons can more safely perform lymphadenectomy in the posterior mediastinum [6]. Some reports have revealed no difference in the blood loss volume, dissection rate, radical resection rate, perioperative complications, and conversion rate, although the operation time is shorter when using the prone position [7].

Kasashima et al. [8] and Kanaji et al. [9] reported that they successfully and safely performed VATS in the prone position for patients with congenital anomalies. However, we believe that the semi-prone position is more beneficial than the conventional prone position because it is easy to change the body position when conversion to open surgery is required in patients with anatomical anomalies, as in our patient. In a search of the PubMed database, we found no previous reports of semi-prone esophagectomy with concomitant ARSA and NRILN. The present report may be the first to describe the accomplishment of semi-prone thoracoscopic esophagectomy for esophageal carcinoma in a patient with ARSA and NRILN.

## Data Availability

Not applicable.

## Abbreviations

ARSA: Aberrant right subclavian artery
NRILN: Non-recurrent inferior laryngeal nerve
RSA: Right subclavian artery
VATS: Video-assisted thoracoscopic surgery
